# Supplementary material for: Strengthening Breast Cancer Screening Mammography Services in Pakistan Using Islamabad Capital Territory as a Pilot Public Health Intervention
Source: Healthcare (Basel). 2022 Jun 14;10(6):1106. doi: 10.3390/healthcare10061106 (PMC9223128; doi:10.3390/healthcare10061106)
Supplement: Supplementary file 1 [file healthcare-10-01106-s001.zip › healthcare-1739277-supplementary.pdf]

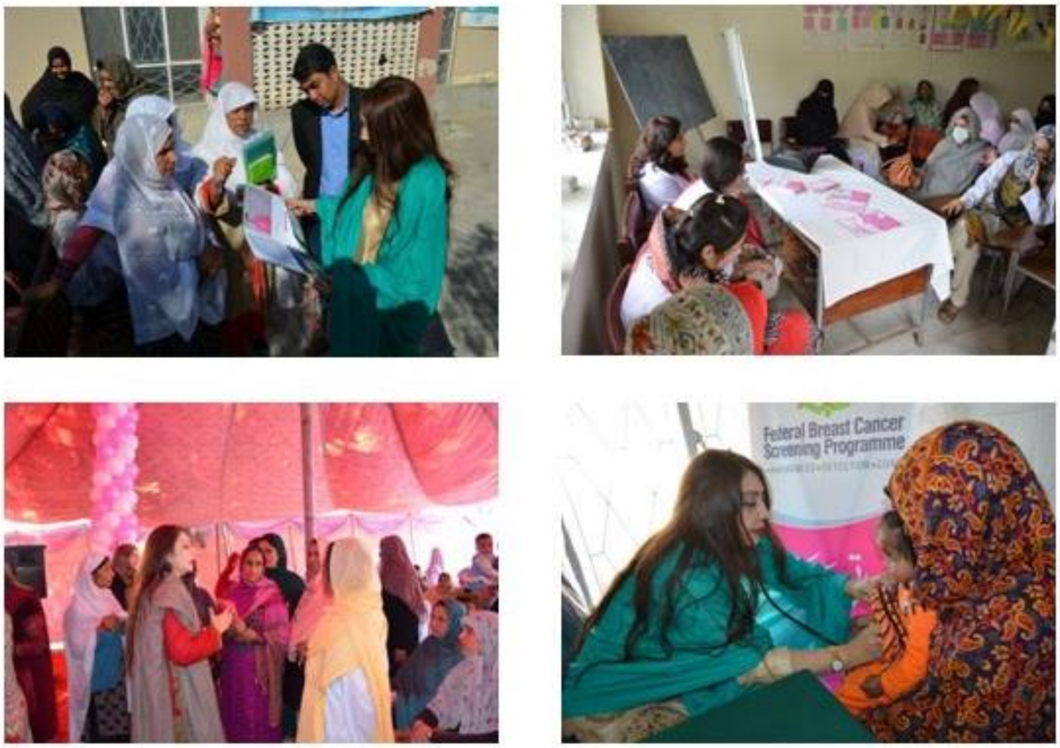

Supplementary Figure S1. **Glimpse of awareness campaigns in rural areas of ICT.**

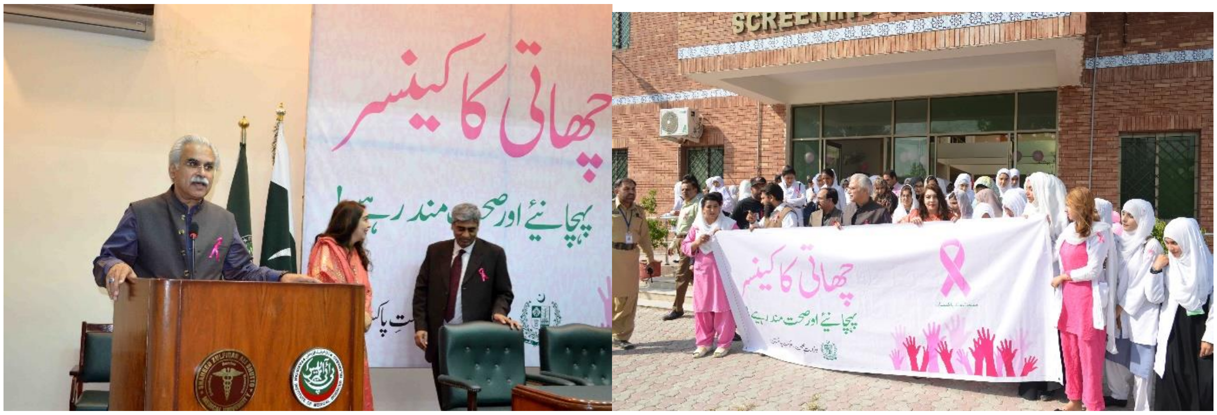

Figure S2. **Workshop for creating awareness regarding Breast Cancer Prevention.**
